# Genetic characterization of Measles Viruses in China, 2004

**DOI:** 10.1186/1743-422X-5-120

**Published:** 2008-10-20

**Authors:** Yan Zhang, Yixin Ji, Xiaohong Jiang, Songtao Xu, Zhen Zhu, Lei Zheng, Jilan He, Hua Ling, Yan Wang, Yang Liu, Wen Du, Xuelei Yang, Naiying Mao, Wenbo Xu

**Affiliations:** 1 WHO WPRO Regional Reference Measles Lab, National Institute for Viral Disease Control and Prevention, China Center for Disease Control and Prevention, Beijing 100050, PR China; 2 Shanxi Provincial Center for Disease Control and Prevention, PR China; 3 Sichuan Provincial Center for Disease Control and Prevention, PR China; 4 Chongqing Provincial Center for Disease Control and Prevention, PR China; 5 Liaoning Provincial Center for Disease Control and Prevention, PR China; 6 Tianjin Provincial Center for Disease Control and Prevention, PR China; 7 Guizhou Provincial Center for Disease Control and Prevention, PR China; 8 Pediatric Institute of People's Hospital of Xinjiang Uygur Autonomous Region, Urumqi city, Xinjiang province, PR China; 9 State Key Laboratory for Molecular Virology & Genetic Engineering, National Institute for Viral Disease Control and Prevention, China Center for Disease Control and Prevention, Beijing 100050, PR China

## Abstract

Genetic characterization of wild-type measles virus was studied using nucleotide sequencing of the C-terminal region of the N protein gene and phylogenetic analysis on 59 isolates from 16 provinces of China in 2004. The results showed that all of the isolates belonged to genotype H1. 51 isolates were belonged to cluster 1 and 8 isolates were cluster 2 and Viruses from both clusters were distributed throughout China without distinct geographic pattern. The nucleotide sequence and predicted amino acid homologies of the 59 H1 strains were 96.5%–100% and 95.7%–100%, respectively. The report showed that the transmission pattern of genotype H1 viruses in China in 2004 was consistent with ongoing endemic transmission of multiple lineages of a single, endemic genotype. Multiple transmission pathways leaded to multiple lineages within endemic genotype.

## Background

Measles virus (MV) is highly contagious and causes a disease characterized by high fever, cough, coryza, conjunctivitis and appearance of a maculopapular rash [1]. It is estimated that measles still causes 345,000 deaths worldwide per year, one-third of all vaccine-preventable childhood deaths [2-4]. However, measles has been eliminated in countries that have maintained high vaccine coverage rates, and four of six WHO regions now have measles elimination goals[5,6]. Other 2 WHO regions now have measles mortality reduction goals.

The WHO Measles and Rubella laboratory Network (LabNet) has been established to monitor progress toward mortality reduction and elimination of measles. The LabNet has grown to include approximately 700 labs in 166 countries confirming measles and rubella cases by IgM testing. Besides serologic testing, another important function of the network is to support the genetic characterization of currently circulating measles viruses. Virological surveillance data, when analysed in conjunction with standard epidemiologic data, can help to document viral transmission pathways and aid in case classification, thus enhancing control programs [7-10]. Molecular epidemiologic data often provides important information for documenting the elimination of endemic transmission of measles. To facilitate virological surveillance, LabNet has standardized the nomenclature and laboratory procedures that are used to describe the genetic characteristics of wild-type measles viruses[11]. WHO currently recognizes 23 genotypes of measles virus [11-15].

China measles lab network was set up in 2001, composed by one national measles lab, 31 provincial measles labs and 331 prefecture labs. Measles virology surveillance had made a great progress. Analysis of wild-type MV circulating in China during 1993–1995 and 1998–1999 led to the identification of a new clade, H [16,17]. Molecular epidemiology of measles viruses in China, 1995–2003 demonstrated that genotype H1 was widely distributed throughout the country and that China has a single, endemic genotype. However, continued sampling of measles virus strains from the different locations around China is needed for a more complete understanding of their evolving in global distribution. We carried out this study to describe the measles genotype circulating in China in 2004 and to complement the database of genetic characteristics of China measles strains during the control phase of the disease.

## Results

**Figure 1:**
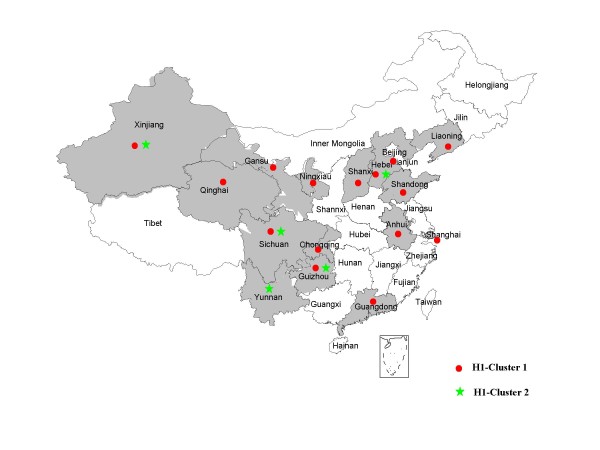
**The geographic distribution of Chinese measles isolates in 2004.** No isolates were received from provinces in white.

59 viral isolates were available from 16 provinces of China (Table 1 and Fig 1). PCR products of the 59 viral isolates in the COOH-terminus of the nucleoprotein gene were available and then sequenced.

**Table 1:** Number of wild-type measles viruses in 2004 by province.

Class*	Province	No. of isolates	Genotype	
			H1	
			
			cluster1	cluster2
A	Guangdong	1	1	0
	Liaoning	5	5	0
	Shanxi	12	12	0
	Tianjin	5	5	0
	Anhui	2	2	0
	Hebei	2	1	1
	Shanghai	2	2	0
	Shandong	2	2	0
B	Chongqing	5	5	0
	Guizhou	4	1	3
	Qinghai	2	2	0
	Xinjiang	3	2	1
	Yunnan	2	0	2
	Gansu	2	2	0
	Sichuan	8	7	1
	Ningxia	2	2	0

	total	59	51	8

Epidemiologic classification of each province is shown

* See definition of epidemiologic class in the text

**Figure 2:**
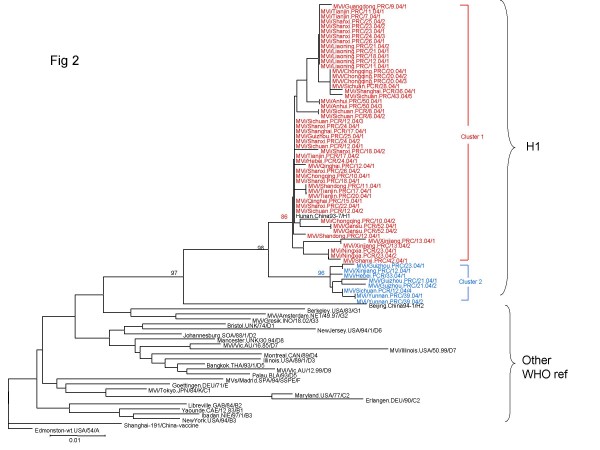
**phylogenetic tree of the N gene sequences of 59 wild-type measles isolates from China compared to the WHO reference sequences for each genotype.** The WHO reference strains and china vaccine, Shanghai-191 were shown in black. Cluster 1 was shown in red, while cluster 2 was shown in blue. WHO strain name is indicated for each sequence.

All of 59 measles isolates in this study clustered within genotype H1. The results of the phylogenetic analysis of carboxyl-terminal coding region of the nucleoprotein (N) gene, of 59 measles isolates in this study, together with the WHO reference strains were shown in Fig 2. The clustering of measles viruses in China 2004 within the genotype H1 was supported by a significant bootstrap value (98% for 1000 replicates). The geographic distributions of genotypes of China isolates are shown in Fig 1. The phylogenetic analysis of all the 59 H1 measles isolates in 2004 illustrated much more complexities involved in the transmission and circulation of H1 genotype measles strain in China. For example, there were identical isolates circulating in different provinces in the same epidemic month; In contrast, identical sequences were sometimes detected during different epidemic month in the same province. 59 H1 isolates were divided into 2 different cluster, 1 and 2. 51 isolates were belonged to cluster 1 and 8 isolates were cluster 2, both of them distributing countrywide without distinct geographical regions.

All genetic changes in the contemporary china isolates evaluated in this study were base substitutions, and no deletion, insertions, or frame-shift mutations. The nucleotide sequence and amino acid homologies of 59 H1 isolates were 96.5%–100% (0–16 nucleotide variation) and 95.7%–100%, respectively. Comparing with WHO H1 genotype reference strain, the nucleotide sequence and amino acid homologies of 59 2004 H1 isolates were 97.7%–100% and 97.2%–100%, respectively.

## Discussion

Measles vaccine was first used in China in 1965, and has been administered routinely to all infants since the China Expanded Programme on Immunization was established in 1978[19]. With the attainment of Universal Childhood Immunization goals, measles mortality and morbidity in China reached lows. During 1995–2004, the incidence of measles was <8/10,000 population, with fewer than 250 measles deaths reported each year[20]. However, outbreaks of measles continue to occur due to accumulation of susceptible children, especially in areas of lower routine immunization coverage. China has made great progresses in measles control and there were some characters of measles epidemic in China. For example: the traditional epidemiology characterization had changed in recent years, that is, the season distribution was delayed and the age distribution was changed; there was great difference among different provinces on the incidence of measles; Outbreak was still the main form of measles in China, the cases of measles outbreaks in 5–10% counties were half of the total measles cases; Floating people were the most risk population due to measles outbreak in the cities. The measles sporadic cases in cities increased, most of them were <8 months children and young adults. All the provinces were divided into 2 groups based on average annual measles incidence: group A and group B[20]. Compared with previous years, more isolates were available from group B provinces in 2004, such as Gansu, Ningxia, Yunnan, Guizhou, which were western poor provinces.

This study included 59 isolates from outbreak or sporadic cases from 16 provinces in 2004. WHO measles network set up the criterion for the specimen collection, that is, in areas that were in the measles elimination phase, the goal would be to obtain appropriate specimens from each chain of transmission; and in areas that were in the measles control and mortality reduction phase, representative samples should be obtained from outbreaks [12,21]. China is now in the phase of accelerated measles control and different provinces were in the different phase of measles control.

The Vero/hSLAM cell line was introduced to China LabNet from 2004. Vero/hSLAM cells are Vero cells that are transfected with a plasmid encoding the gene for the human SLAM molecule (Ono, et al., 2001). Vero/hSLAM cells are able to bind to both wild type isolates and laboratory adapted strains of measles viruses, and this cell line has been recommended for use in the WHO measles and rubella laboratory network.

Genetic analysis results showed that the H1 genotype virus was still the predominant endemic measles virus in China in 2004. H1 genotype measles was also detected epidemic in Japan, Korea [22-24]. But except for H1 genotype, there was D3, D5 and D9 genotypes epidemic in Japan. And in the neighboring country of China, there were different genotypes epidemic, such as D4, D8 in Nepal, D4 in Pakistan, G2 in Thailand, H2 in Vietnam. In the west neighboring European country, there is still country with no report of genotype information [15]. Monitoring the pattern of measles genotypes in an area can help document the effectiveness of control measures. In China, which still have endemic transmission of measles, virologic surveillance of cases detects a limited number of genotypes, and Cambodia, Turkey, Vietnam has the same situation [17,25,26]. On the other hand, in areas where endemic transmission of virus has been interrupted, a variety of genotypes are detected, reflecting the multiple sources of imported viruses, such as USA, Australia, Canada and the United Kingdom [8,27-29]. Since WPRO, including China, has recently initiated a program to eliminate measles in 2012, maybe a variety of genotypes will be detected in China as the intensity of the measles control and frequent travel communication between different countries. H1 also imported to USA from China between 1999 and 2005.

The phylogenetic tree of 59 H1 isolates showed that evidences for multiple chains of transmission. There were sustained chains of transmission in most of provinces. Outbreak was the main form of measles in China. The identical wild-type measles virus strain could induce outbreaks in different epidemiologic month in different provinces, maybe these outbreaks were caused by identical wild-type measles viruses transmitting among different provinces for several months and there was a mutual transmission between provinces in different months.

Single endemic H1 isolates formed two clusters, cluster 1 and cluster 2. Cluster 1 is the predominant cluster circulating in China in 2004. There were multiple lineages in each cluster. These data reinforce the observation that multiple chains of transmission were present in areas that had endemic measles. The transmission pattern of genotype H1 viruses in China in 2004 was consistent with ongoing endemic transmission of multiple lineages of a single, endemic genotype. Multiple transmission pathways leaded to multiple lineages within endemic genotype(s).

## Conclusion

This study reports virologic surveillance data obtained in 16 provinces of China during 2004. The results confirmed that genotype H1 is the endemic genotype circulating in at least 16 provinces of China. The virologic data were consistent with endemic measles in that multiple chains of transmission were evident. The H1 viruses were very diverse and formed two major clusters, which were distributed throughout 16 provinces with no apparent geographic restriction. This important baseline data contribute to the development of improved measles control programs in China.

## Methods

### Specimens collection and virus isolation

Throat swab and urine samples were obtained from serologically confirmed measles cases. Clinical specimens were inoculated onto B95a cells or Vero/SLAM (signaling lymphocyte-activation molecule; also known as CDw150) cells [18], and the cells were observed for cytopathic effect (CPE). Inoculated cells were blind-passaged up to three times before being discarded. Cells were harvested when the CPE was maximal. Virus isolation was performed by 16 provincial laboratories in China and the viral isolates were shipped to the National Measles Laboratory, in Beijing for genetic analysis.

### RNA Extraction and RT-PCR

Viral RNA was extracted from infected cell lysates using Trizol reagent according to the manufacturer's directions. RNA pellets were dried and resuspended in 50 μl of sterile distilled water and stored at -70 C until amplification by RT-PCR. RT-PCR was performed using previously described methods [6,20]. Primers MV63 (5'CCT CGG CCT CTC GCA CCT AGT 3') and MV60 (5'GCT ATG CCA TGG GAG TAG GAG TGG 3') were used to amplify a 676 bp fragment of the N gene including the 450 bp fragment recommended for genotyping.

### Sequence analysis

The sequences of the PCR products were derived by automated both strands sequencing with primers MV60 and MV63 and the BigDye terminator v2.0 chemistry using reaction conditions that were recommended by the manufacturer (ABI 373, ABI 3100, Perkin Elmer-Applied Biosystems). Sequence proof reading and editing was conducted with Sequencer™ (Gene Codes Corporation). Sequence data were analyzed by using version 7.0 of Bioedit and phylogenetic analyses were performed using Bioedit and Mega ver3.1. The robustness of the groupings was assessed using bootstrap resampling of 1000 replicates and the trees were visualized with Mega programs. 45 representative nucleotide sequences were deposited in GenBank under accession numbers: EU557194–EU557238.

## Abbreviations

MV: Measles virus; RT-PCR: reverse transcriptase polymerase chain reaction; H: Hemagglutinin; N: Nucleoprotein; WHO: World Health Organization.

## Competing interests

The authors declare that they have no competing interests.

## Authors' contributions

YZ, WBX prepared manuscript. WBX designed the study and organized the coordination. YZ performed RT-PCR, sequence and data analysis. YZ, YXJ, STX, ZZ, NYM performed RT-PCR and sequence analysis. XHJ, LZ, JLH, HL, YW, YL, WD and XLY collected specimens and performed virus isolation, viral identification. All authors read and approved the final manuscript.
